# Abstract of Meteorological Observations for May, 1854, Made at Philadelphia, Pa.

**Published:** 1854-07

**Authors:** James A. Kirkpatrick


*Abstract of Meteorological Observations for May,* 1854, *made at Philadelphia, Pa. Latitude* 39° 57′ 28″ *N., Longitude* 75° 10′ 40″ *W. from Greenwich.* By PROF. JAMES A. KIRKPATRICK.

| 1854. May. | Barometer. Daily Mean | Barometer. Mean Daily Range. | Thermom. Daily Mean | Thermom. Mean Daily Range | Dew Point 2 P.M. | Rain. | Prevailing Winds. | General Remarks. |
|---|---|---|---|---|---|---|---|---|
| | Inches. | Inches. | Deg. | Deg. | Deg. | Inch. | Points. | |
| 1 | 29.833 | .018 | 49.5 | 2.3 | 44.0 | | SW. | Clear. |
| 2 | 29.767 | .112 | 59.5 | 9.0 | 44.0 | 0.391 | SW. | Cloudy. |
| 3 | 29.630 | .136 | 50.0 | 11.2 | 47.3 | 0.217 | W. | Cloudy. *Barom. lowest* 29.585 in. |
| 4 | 29.698 | .072 | 53.5 | 5.5 | 46.0 | 0.303 | N. | M. and aft. cloudy; ev. clear. |
| 5 | 29.673 | .082 | 60.0 | 8.5 | 48.7 | 0.018 | SW. | Clear. Aft. showers. |
| 6 | 29.682 | .041 | 54.5 | 13.5 | 39.8 | | (Var.) | M. cloudy; aft. and ev. clear. |
| 7 | 29.733 | .073 | 46.0 | 8.7 | 26.7 | | W. | Clear. *Therm lowest* 35°. |
| 8 | 29.741 | .044 | 54.7 | 7.5 | 33.8 | | NW. | Clear. |
| 9 | 29.753 | .030 | 61.0 | 7.2 | 40.0 | | SW. | Aft. clear; m. and ev. cloudy. |
| 10 | 29.724 | .042 | 71.0 | 6.0 | 58.7 | 0.435 | SE. | Cloudy. Thunder storm with hail. |
| 11 | 29.804 | .091 | 68.5 | 3.2 | 50.7 | 0.040 | SW. | Cloudy. |
| 12 | 30.036 | .231 | 67.5 | 1.5 | 46.7 | | (Var.) | Clear. |
| 13 | 30.135 | .099 | 68.0 | 2.0 | 56.3 | 0.011 | ESE. | M. and aft. cloudy; ev. clear. *Barom.* |
| 14 | 30.033 | .102 | 68.5 | 2.0 | 69.0 | 0.851 | (Var.) | Cloudy. [*highest* 30.153 in. |
| 15 | 29.916 | .083 | 71.5 | 3.0 | 64.0 | | SW. | M. and aft. cloudy; ev. clear. |
| 16 | 29.949 | .039 | 71.5 | 1.5 | 47.3 | 0.359 | NE. | Clear. Dur. night of 15th, *rain, hail,* |
| 17 | 29.859 | .101 | 73.0 | 1.5 | 56.3 | 0.761 | SSE. | Cloudy. [*thunder and lightning.* |
| 18 | 29.730 | .129 | 75.0 | 2.3 | 58.7 | 0.024 | SW. | Cloudy. |
| 19 | 29.890 | .161 | 69.0 | 3.7 | 47.7 | | SW. | Clear. |
| 20 | 29.930 | .039 | 69.5 | 1.5 | 41.7 | | (Var.) | Clear. |
| 21 | 29.952 | .037 | 61.5 | 6.8 | 59.3 | 0.434 | (Var.) | M. and aft. cloudy; ev. clear. |
| 22 | 30.005 | .054 | 66.0 | 4.7 | 47.3 | | NE. | Clear. |
| 23 | 30.117 | .112 | 62.0 | 3.0 | 37.3 | | NE. | Clear. |
| 24 | 30.070 | .047 | 66.0 | 4.0 | 43.3 | | (Var.) | M. and aft. cloudy; ev. clear. |
| 25 | 29.815 | .255 | 65.0 | 4.7 | 68.7 | 2.548 | SW. | Cloudy. |
| 26 | 29.759 | .079 | 69.5 | 5.7 | 42.0 | | NW. | Clear. |
| 27 | 29.804 | .078 | 66.0 | 3.3 | 49.3 | | NW. | Clear. |
| 28 | 29.824 | .032 | 71.0 | 3.7 | 53.0 | | (Var.) | Clear. |
| 29 | 29.778 | .051 | 73.5 | 2.5 | 51.7 | | ENE. | Cloudy. *Therm. highest* 84°. |
| 30 | 29.687 | .086 | 72.5 | 2.2 | 62.3 | 0.907 | NE. | Cloudy. |
| 31 | 29.953 | .266 | 57.0 | 15.5 | 40.0 | | (Var.) | Cloudy. |
| Means for 1854 | 29.847 | .091 | 64.3 | 5.1 | 49.1 | 7.299 | S85°W14-100 | |
| Means for 1853 | 29.888 | | 63.1 | | 49.3 | 5.170 | | |
| Means for 1852 | 29.889 | | 64.1 | | 48.5 | 3.040 | | |
| 3years | 29.875 | | 63.8 | | 49.0 | 5.170 | S86½W33.100. | |

The observations are made at 7 A.M., 2 P.M. and 9 P.M. The barometric mean is found by combining the three observations; the thermometric mean by combining the *lowest* for the day with the temperature at 2 P.M. The Dew Point is found by calculation from Mason's Hygrometer. The mean daily range of the barometer and thermometer is found by taking the mean of the differences between the observations of one and those of the corresponding hours of the preceding day. The barometric observations have all been corrected for temperature. The extreme range of the barometer during the month was 0.568 of an inch, and of the thermometer 49°. The resultant of the winds for the month is found by Schow's method.

